# The dataset on rural women’s awareness and attitudes about residential constructions in accordance with the health standards A case study of Gilan-e-Gharb, Iran

**DOI:** 10.1016/j.dib.2018.08.078

**Published:** 2018-08-29

**Authors:** Yaya Safari, Nasrin Yoosefpour, Mohammad Darvishmotavalli, Yasser Vasseghian, Kamaladdin Karimyan, Vinod Kumar Gupta, Omid Nasri, Arash Ziapour

**Affiliations:** a Research Center for Environmental Determinants of Health (RCEDH), Kermanshah University of Medical Sciences, Kermanshah, Iran; b Students Research Committee, School of Public Health, Kermanshah University of Medical Sciences, Kermanshah, Iran; c Environment Research Center, Isfahan University of Medical Sciences, Isfahan, Iran; d Environmental Health Research Center, Kurdistan University of Medical Sciences, Sanandaj, Iran; e Department of Environmental Health Engineering, School of Public Health, Tehran University of Medical Sciences, Tehran, Iran; f Department of Applied Chemistry, University of Johannesburg, Johannesburg, South Africa

**Keywords:** Awareness, Attitudes, Rural women, Residence, Gilan-e-Gharb Township

## Abstract

Residence can affect various aspects of one׳s physical, psychological and social health. Therefore, the present dataset aimed to assess the level of rural housewives’ awareness and attitudes towards the importance of residence and its compliance with health standards. To collect the present dataset, four villages were selected from the Gilan-e-Gharb township using the randomized cluster sampling method, then 25 subjects were chosen from each village (totaling 100 altogether). Furthermore, the subjects’ awareness and attitudes were measured using a researcher-made questionnaire, and the data were then analyzed using the SPSS Statistical Software Version 21.0. The obtained data demonstrated that rural housewives’ awareness and attitudes towards the subject were significantly different in terms of education and age group (*P*< 0.05), but the opposite was true in terms of the variables of marital status and training by health practitioners (*P*> 0.05). Based on the obtained data, the awareness and attitudes of rural women towards the importance and necessity of health standards of residence were low and moderate, respectively. In addition, providing rural women with effective training in various ways to raise their awareness and attitudes is of prime significance.

**Specifications Table**

**Table:** 

Subject area	Environmental health
More specific subject area	Housing health
Type of data	Tables
How data was acquired	Four villages were selected from the Gilan-e-Gharb township using the randomized cluster sampling method, then 25 women were chosen from each village (totaling 100 altogether). The women’ awareness and attitudes were measured using a researcher-made questionnaire.
Data format	Raw, analyzed
Experimental factors	All questions were scored on a Likert Scales. Unanswered questions or invalid answers were regarded as missing data and excluded. The validity and reliability of the questionnaire were evaluated using content validity and test-retest, respectively.
Experimental features	To compare the means of two groups of variables and more, the independent sample t-test and ANOVA were used, respectively.
Data source location	Gilan-e-Gharb Township, Kermanshah Province, Iran
Data accessibility	Data were included in this article
Related research article	Y. Safari, K. Karimyan, V.K. Gupta, A. Ziapour, M. Moradi, N. Yoosefpour, M. Akhlaghi, H. Sharfi, A Study of Staff׳s Awareness and Attitudes towards the Importance of Household Hazardous Wastes (HHW) Management (A Case Study of Kermanshah University of Medical Sciences, Kermanshah, Iran), Data Brief. 19(2018) 1490–1497 [1].
	Y. Safari, S. Maleki, K. Karimyan, H. Arfaeinia, V.K. Gupta, N.Yoosefpour, N. Shalyari, M. Akhlaghi, H. Sharfi, A. Ziapour, Data for interventional role of training in changing the knowledge and attitudes of urban mothers towards food hygiene (A case study of Ravansar Township, Kermanshah, Iran), Data brief. 19(2018) 67–75 [2].

**Value of the Data**

• Awareness of the health standards of residence in term of different aspects is necessary for everyone in society [3], [4], [5], [6], [7]. Accordingly, to determine the level of said awareness, some methodologies and measurement tools are required, which were used in the present dataset. In addition this can be useful for future similar studies.
• Limited studies have been conducted on the subject under discussion [8], [9], [10]. Therefore, the obtained data from present dataset can be used as the basis for future studies.
• Due to the lack of previous information in this respect, the obtained data is useful for improving the health status of residential constructions in this region.
• The data can be used by health authorities and decision-makers in the relevant area.

## 1 Data

According to the demographic data of the subjects, the rural housewives were mostly married, in the 21–40 age range, holding high school diplomas, and trained by health practitioners (Table 1).

**Table 1:** The demographic data of the subjects under study.

**Variables**		**Frequency**	
		**Number**	**Percentage**
Marital status	Single	25	25
	Married	75	75
Have they been trained by health practitioners?	Yes	68	68
	No	32	32
Education	Primary school	11	11
	Middle school	14	14
	Secondary school	49	49
	University	26	26
Age range (in years)	< 20	12	12
	21–40	54	54
	41–60	34	34

**Fig. 1:**
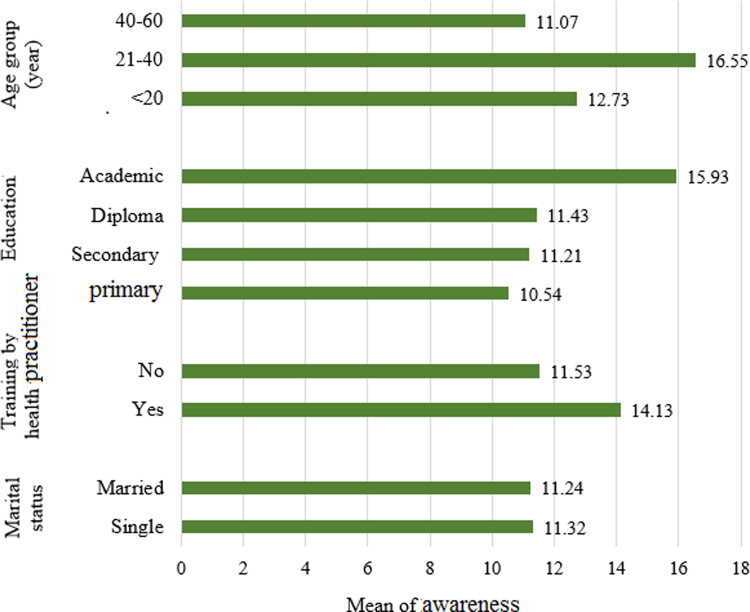
The data of comparing the means of overall awareness in terms of various variables.

The data revealed that the awareness of the subjects under study was average in terms of items 4, 5, 7, and 8, as opposed to high and very high in terms of other items (Table 2). In Fig. 1, the data of comparing the means of overall awareness in terms of various variables are shown.

**Table 2:** The scores obtained by the subjects on each component of awareness.

**Awareness**			**Level**
**Component number**	**Components**	**Score (Maximum)**	
1	Housing safety	1.15 (2)	High
2	Waste management at home	1.76 (6)	High
3	Housing lighting and its importance	2.34 (3)	Very high
4	The housing structure and its affordable space	1.44 (4)	Average
5	suitable temperature of the residence and its importance	0.35 (1)	Average
6	The color of walls and ceiling and its importance	0.8 (1)	Very high
7	The importance of indoor air pollution and its ventilation	1.27 (4)	Average
8	Other related topics and general concepts	0.97 (3)	Average

**Fig. 2:**
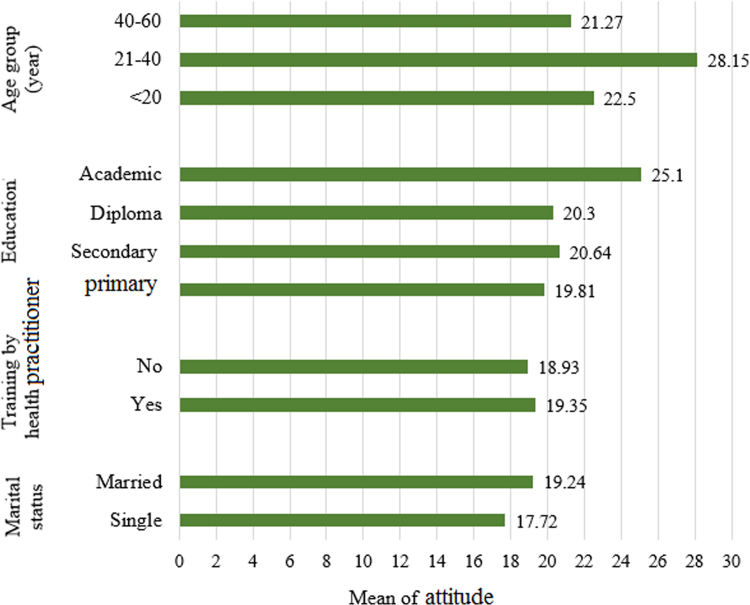
The results of comparing the means of overall attitude in terms of various variables.

The obtained data demonstrated that the attitude of the subjects under study was low and average in terms of eight items (Table 3). In Fig. 2, the data of comparing the means of overall attitude in terms of various variables are shown.

**Table 3:** The scores obtained by the subjects on each component of attitude.

**Attitude**			**Level**
**Component number**	**Components**	**Score (Maximum)**	
1	Housing safety	2.42 (9)	Average
2	Waste management at home	1.76 (6)	Average
3	Housing lighting and its importance	0.82 (3)	Average
4	The housing structure and its affordable space	0.73 (3)	Low
5	The color of walls and ceiling and its importance	1.78 (6)	Average
6	The importance of indoor air pollution and its ventilation	12.3 (33)	Average
7	Other related topics and general concepts	1.96 (15)	Low
8	Noise	1.04 (6)	Low

## 2 Experimental design, materials and methods

### 2.1 Study area

Gilan-e-Gharb Township is geographically located in the west of Kermanshah Province. It has a latitude of 45°, 55 min and 13° and 34 min northern latitude, 91° and 45 min eastern longitude in relation to Greenwich meridian. Gilan-e-Gharb Township, with a 150-km distance from the capital of Kermanshah, borders Sarpol-e zahab and Kerend Townships from the north, Islamabad Gharb from east, Eiwan Gharb, Somar and Mandali Counties from south, and Naftshahr and Ghasr-e Shirin Townships from west. According to the census data of the health center collected in 2014, the population of Gilan-e-Gharb Township numbered 60,435 in 2014. Moreover, there are 181 villages in this township, from which four were randomly selected (Gravian, Sheikh Sorkh al-Din Sofla, Sarab and Sang Kermoo Shirzadi) with a total population of 2670 and 604 households. The number of women in these four villages were 1311 people.

### 2.2 Study design and data collection

To conduct the present descriptive and cross-sectional study, a researcher-made questionnaire was designed, and the statistical population consisted of the rural housewives residing in Gilan-e-Gharb Township. Four villages were selected using the randomized cluster sampling method, and then 25 subjects were chosen from each village (totaling 100 altogether). The required sample size was determined using the formula for determining the sample size with a single population and considering *d* = 0.05 and *α* = 0.05, and the mean and previous variance. Moreover, a researcher-made questionnaire was used to collect raw data, and content validity was applied to measure the validity of the questionnaire. To do so, the intended questionnaire was given to 10 faculty members of the Faculty of Health and 10 employees at the environmental health centers of Kermanshah and Gilan-e Gharb Townships to be examined based on the objectives of the study and the questions relating to attitude and awareness. Furthermore, the test-retest method was used to determine the reliability of the questionnaire [9], [10], [11], [12], [13], [14], [15], [16].

In this test, the questionnaires were first completed by 10 rural women. Then, the same subjects were retested, and the reliability of the questionnaire was evaluated using the Pearson correlation coefficient, which measured 0.8 and 0.7 for questions on awareness and attitude, respectively. The questionnaire was arranged in three sections. The first part consisted of demographic information, the second part consisted of 23 questions about awareness (with one point one for each one), and the third part consisted of 27 questions about attitude. After collecting the questionnaires, the data were analyzed using the SPSS Statistical Software Version 21.0. Then, the significance of difference between the mean scores of awareness and attitude was examined between the married and single subjects with and without exposure to training provided by health practitioners. In addition, the significance of difference between the mean scores of awareness and attitude was examined between different age groups and education levels using one-way ANOVA. Finally, the descriptive statistics were presented using descriptive parameters. In Table 4, Table 5, the scores of studying the components of awareness and attitude are shown based on Likert scale, respectively.

**Table 4:** The scores of studying the components of awareness based on likert scale.

**Component number**	**Components**	**Number of questions**	**Maximum score**	**Level of Awareness**			
				**Low**	**Average**	**High**	**Very high**
1	Housing safety	5	5	0–1.24	1.25–2.49	2.5–3.74	3.75–5
2	Waste management at home	2	2	0–0.49	0.5–0.99	1–1.49	1.5–2
3	Housing lighting and its importance	3	3	0–0.74	0.75–1.49	1.5–2.24	2.25–3
4	The housing structure and its affordable space	4	4	0–0.99	1–1.99	2–2.99	3–4
5	Suitable temperature of the residence and its importance	1	1	0–0.24	0.25–0.49	0.5–0.74	0.75–1
6	The color of walls and ceiling and its importance	1	1	0–0.24	0.25–0.49	0.5–0.74	0.75–1
7	The importance of indoor air pollution and its ventilation	5	5	0–0.99	1–1.99	2–2.99	3–4
8	Other related topics and general concepts	3	3	0–0.74	0.75–1.49	1.5–2.24	2.25–3
Overall Awareness		23	23	0–5.99	6–11.99	12–17.99	18–23

**Table 5:** The scores of studying the components of attitude based on likert scale.

**Component number**	**Components**	**Number of Questions**	**Maximum Score**	**Level of Attitude**			
				**Low**	**Average**	**High**	**Very high**
1	Housing safety	3	9	0–2.24	49.5-4.2	74.5-6.	75-9.6
2	Waste management at home	2	6	0–1.49	99.5-2.1	49.3-4	5–6.4
3	Housing lighting and its importance	1	3	0–0.74	49.75-1.0	24.5-2.1	25-3.2
4	The housing structure and its affordable space	1	3	0–0.74	49.75-1.0	24.5-2.1	25-3.2
5	The color of walls and ceiling and its importance	2	6	0–1.49	99.5-2.1	49.3-4	5–6.4
6	The importance of indoor air pollution and its ventilation	11	33	0–8.24	49.25-16.8	74.5-24.16	75-33.24
7	Other related topics and general concepts	5	15	0–3.74	49.75-7.3	24.5-11.7	25-15.11
8	Noise	2	6	0–1.49	99.5-2.1	49.3-4	5–6.4
Overall Attitude		27	81	0–19.99	99.20-39	99.40-59	60–81
